# Does facility readiness promote high-quality of provider-initiated HIV testing and counseling to pregnant women? A national survey for improving policy of prevention of mother-to-child transmission of HIV in Tanzania

**DOI:** 10.1186/s12981-021-00362-y

**Published:** 2021-07-03

**Authors:** Deogratius Bintabara, Athanase Lilungulu, Shakilu Jumanne, Mzee M. Nassoro, Bonaventura C. Mpondo

**Affiliations:** 1 Department of Community Medicine, The University of Dodoma, Dodoma, Tanzania; 2 Department of Obstetrics and Gynecology, The University of Dodoma, Dodoma, Tanzania; 3 Department of Pediatrics and Child Health, The University of Dodoma, Dodoma, Tanzania; 4 Department of Obstetrics and Gynecology, Dodoma Regional Referral Hospital, Dodoma, Tanzania; 5 Department of Internal Medicine, The University of Dodoma, Dodoma, Tanzania

**Keywords:** Facility readiness, High-quality, Antenatal care, Provider-initiated HIV testing and counseling, PMTCT, Tanzania

## Abstract

**Background:**

Provider-initiated HIV testing and counseling (PITC) is a recommended approach to screen for HIV to all pregnant women during antenatal care (ANC) visits, and all with HIV positive results have to be enrolled into prevention of mother-to-child transmission of HIV (PMTCT) program. However, little is known about the relationship between facility readiness and the uptake of PITC to pregnant women attending ANC in Tanzania. Therefore, this study assessed whether the facility readiness promotes the uptake of PITC to the pregnant women attending ANC for the purpose of improving the PMTCT interventions in Tanzania.

**Methods:**

This study analyzed data for health facilities obtained from the 2014–2015 Tanzania service provision assessment survey. The Primary outcome measure was a composite variable (with score of 0–5) in which its higher scores indicates provision of high-quality of PITC. Also, facilities scored higher in the PMTCT service readiness index were considered to have high readiness to provide PMTCT services. In Poisson regression analyses, a series of models were fitted to assess whether there is an association between provision of high-quality of PITC and facility readiness. In all statistical analysis, a *P* < 0.05 was considered significant.

**Results:**

Out of 1853 included first-visit ANC consultations, only about one-third of pregnant women received all five components required for PITC. The mean percentage of PMTCT readiness score was moderate 63.96 [61.32–66.59]%. In adjusted model, we found that facility with high readiness to provide PMTCT services was significantly associated with the provision of high-quality of PITC (model 2: [β = 0.075, *P* = 0.00]).

**Conclusion:**

In order to increase high-quality of PITC services, efforts should be made to improve the PMTCT facility readiness by increasing availability of trained staffs, diagnostic tools, and ARTs among health facilities in Tanzania.

**Supplementary Information:**

The online version contains supplementary material available at 10.1186/s12981-021-00362-y.

## Background

Even though sub-Saharan Africa (SSA) contains only about 11% of the World’s population, the region is home to about 70% of worldwide people infected with HIV [1]. In this region, the main mode of HIV transmission is through heterosexual sex, with women disproportionally affected [1, 2]. It is estimated that 95% of all HIV positive women and 80% of the 3,80,000 young women who are newly infected with HIV live in SSA [3, 4]. Besides, as the pregnancy status increases the risk of acquiring HIV infection [5], thisplacewomen in SSA which is the area with high fertility rate and burden of sexually transmitted diseases to be at most vulnerable for HIV infection [6]. Hence, in the absence of any preventive measures, about 25–40% of HIV-positive women will transmit the virus to their newborns [7]. The rates of Mother-To-Child Transmission (MTCT) is relatively high in SSA with approximately 1000 newborns infected every day [8, 9].

In Tanzania, MTCT accounts for about 18% of new HIV infections and approximately 99,000 HIV-positive women are estimated to deliver exposed infants annually [10]. To eliminate this burden of MTCT, in the early 2000s, the Tanzanian government established and then scaled up the program for the Prevention of Mother-to-Child Transmission of HIV (PMTCT) as suggested by World Health Organization (WHO) for the countries with limited-resource [11, 12]. The package of PMTCT program has been integrated into routine Reproductive and Child Health (RCH) services to ensure that there is a reduced vertical transmission [12]. Similar to other SSA countries, Tanzania made tremendous progress in coverage and accessibility of PMTCT services. However, the country faces the challenge to enroll all HIV-positive pregnant women due to inadequate uptake of HIV testing and counseling [13]. This slow the efforts toward achieving the elimination of MTCT (EMTCT) goal, defined as a reduction of the number of new HIV infections occurring during MTCT by 90%, reduction in the final MTCT rate to ≤ 5% among breastfeeding, and ≤ 2% among non-breastfeeding populations [14, 15]. Until 2016, Tanzania achieved 69% MTCT reduction and the MTCT rate at the end of breastfeeding gone down to 8% [16].

The cascade of PMTCT services in Tanzania starts with provider-initiated HIV testing and counseling (PITC) during antenatal care (ANC) attendance [17]. Through PITC all pregnant women at the first-visit of ANC or follow-up visits are expected to receive HIV testing and counseling, those diagnosed with HIV are supposed to be enrolled into the PMTCT program as early as possible [18]. Although about 98% of pregnant women attended ANC at least once [19], not all received PITC services. This affect the enrollment of HIV positive pregnant women into the PMTCT program and subsequently missed opportunities for the timely initiation of treatment with antiretroviral therapy [17].

The limited information regarding the observed discrepancy between high proportions of women attending to ANC with the availability of PMTCT service and the poor provision of high-quality of PITC prompted researches on this area. Some of the researches aimed to identify factors associated with poor uptake of PITC at multiple levels such as poor knowledge of HIV vertical transmission, age, maternal education, psychological problems after HIV diagnosis, stigma and fear of status disclosure to partners, family or community members. Other reported factors were poor staff-patient interactions, staff shortages, service accessibility, and home deliveries [20, 21]. Despite the efforts of these researchers to identify those factors, no one examined the level of facility readiness to enhance the provision of high-quality of PITC for PMTCT.

Facility readiness defined as “willingness or capacity of health facility to provide PITC services for the PMTCT” is important to evaluate how the facility is committed to fighting against MTCT of HIV infection. For that reason, not only availability of services but also together with facility readiness will promise patient to receive high-quality of care [22, 23]. Therefore, a full understanding of the relationship between facility readiness and provision of high-quality of PITC to pregnant women attending ANC is crucial as little is known about this relationship. The current study was conducted to assess whether the facility readiness associated with provision of high-quality of PITC to pregnant women attending first-visit ANC in Tanzania.

## Methods

### Data source

This study analyzed data from the 2014–2015 Tanzania Service Provision Assessment (TSPA) survey. TSPA was designed to assess all health facilities in Tanzania [24]. The survey provides information on the availability of basic and essential health care services and service readiness. One of the issues the survey assessed is the presence and functions of components essential for high-quality service delivery for antenatal care services including HIV counseling and testing services as a package of PMTCT.

### Study sample and sampling procedure

**Fig. 1:**
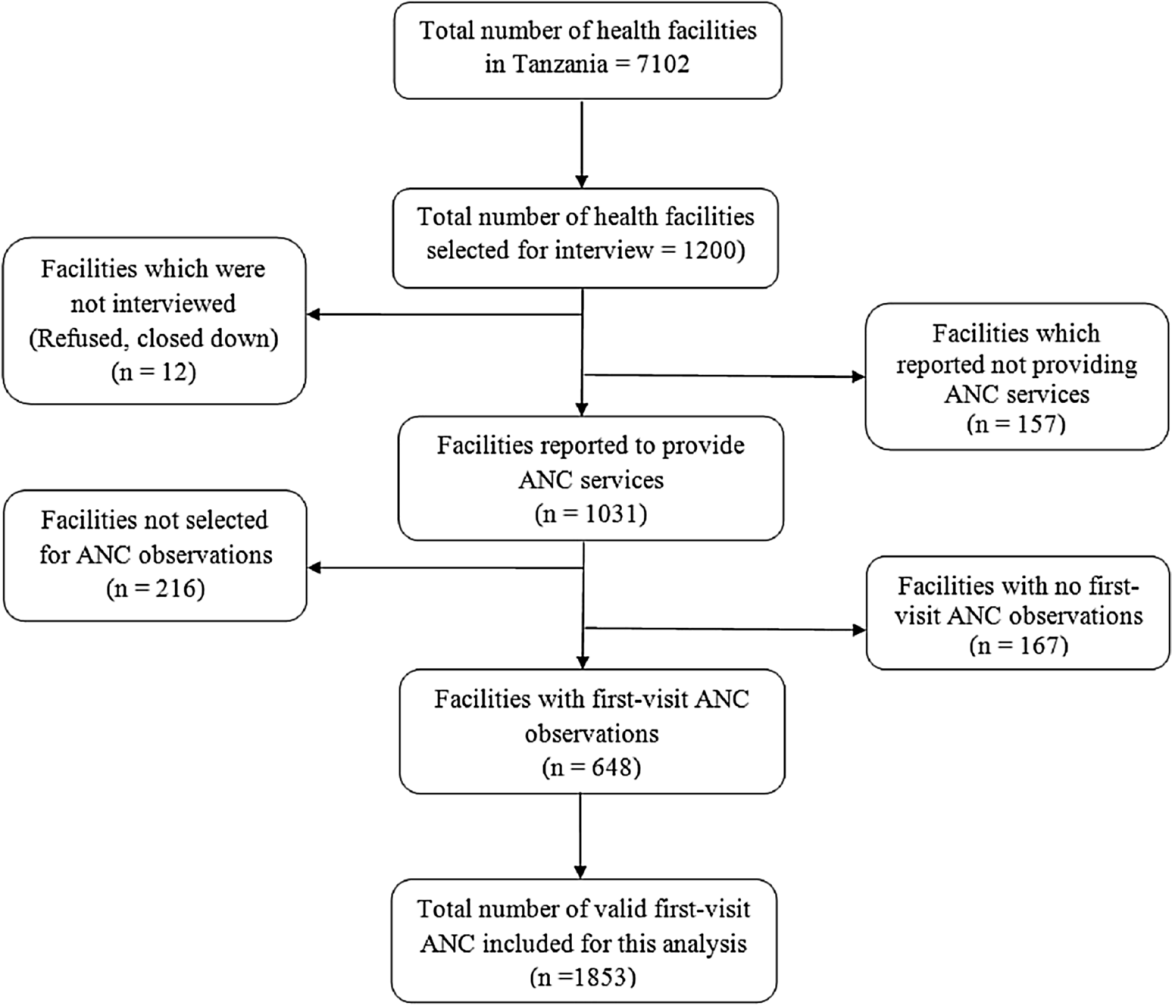
Selection procedure of the health facilities and ANC visits included in this analysis

A list of all the formal sector health facilities such as hospitals, health centres, dispensaries, and clinics provided by the Ministry of Health and Social Welfare (MoHSW) in Tanzania Mainland and the Ministry of Health (MOH) in Zanzibar was used as a sampling frame in which a sample of 1200 facilities was selected to participate in the survey. More details about TSPA survey sampling procedures are available online [24]. Based on the objectives of this research, the current analysis was restricted to health facilities with at least one observed first-visit ANC consultation. Hence, facilities that reported providing ANC services, selected to participate for ANC observation, were open on the day of the interview, and agreed to participate were eligible and were selected in this study. Facilities that did not meet these criteria were excluded from the study (seven refused to participate, four were closed on the day of the interview, one could not be reached, 157 did not provide ANC services, 216 were not selected for ANC observation, 167 did not have first ANC visit observations). Therefore, after excluding all facilities that did not meet the inclusion criteria, a total of 648 health facilities in which 1853 first-visit ANC consultations were performed were included for this analysis (Fig. 1).

During data collection, the interviewers used the Observation Questionnaire to assess whether the processes followed in observed client-provider consultations met standards for acceptable content and quality during service delivery. In this case, the interviewers acting as observers sat in on consultations for ANC services. They recorded the information shared between client and provider and the processes the provider followed when assessing the client, conducting procedures, and providing treatment. In our analysis, we focused only on the provision of PITC related services for the PMTCT.

### Measurement of variables

#### Outcome variable

“Provision of high-quality of PITC,” was a composite score created by using the responses observed by interviewers if the provider performed the following five important services of PITC to the pregnant women during first-visit ANC consultations. These included (i) establishing client’s HIV status, (ii) provide or refer for counseling related to HIV test, (iii) perform or refer for HIV test, (iv) provided post-test counseling and v) discussed partner testing. Each of these services scored “1” if the provider was observed to perform it, otherwise scored “0.” The composite score ranges from 0 to 5. The higher scores regarded as provision of high-quality of PITC during first-visit consultation compared to the lower scores.

#### Key independent variable

Facility readiness; in this study was measured based on the score of PMTCT specific services readiness index. This score was determined using a WHO approach, while the PMTCT readiness indicators were identified according to WHO Service Availability and Readiness Assessment (WHO-SARA) reference manual [25]. Using this approach, the PMTCT service readiness index was categorized into four domains. The first domain was “staff and guidelines,” which had four indicators, i.e., guidelines for PMTCT, guidelines for infant and young child feeding counseling, staff trained in PMTCT, and staff trained in infant and young child feeding. The facilities that reported having guidelines for PMTCT or guidelines for PMTCT and infant and young child feeding counseling was categorized as “Yes,” while those without such guidelines were categorized as “No.” Also, facilities with at least one staff member that had received refresher training in PMTCT and infant and young child feeding counseling within two years before the interview was categorized as “Yes,” while those without such staff members were categorized as “No.” The second domain was equipment, which had one indicator, i.e., the presence of visual and auditory privacy. Facilities with the private room or screened off area for PMTCT that a normal conversation can be held without being overheard, and without the client being observed were categorized as “Yes,” while those without were categorized as “No.” The third domain was diagnostics, which had two indicators, i.e., HIV diagnostic capacity for adults and HIV diagnostic capacity for infant/ young child. Facilities with HIV Rapid Diagnostic Test (RDT) or ELISA for HIV testing of adults were categorized as “Yes,” while those without were categorized as “No.” in addition, facilities with Dried Blood Spot (DBS) filter paper for diagnosing HIV in newborns were categorized as "Yes," while those without were categorized as "No." The fourth domain was medicine and commodities which has three indicators, i.e., the availabilities of Zidovudine syrup, Nevirapine syrup, and Maternal Antiretroviral (ARV) prophylaxis (either Option A: AZT, NVP, and 3TC or Option B: AZT + 3TC + LPV or AZT + 3TC + ABC or AZT + 3TC + EFV or TDF + 3TC (or FTC) + EFV). Each of the medicine indicators was categorized as “Yes” for the facilities reported the availability of that medicine(s) and otherwise was categorized as “No.” For details see Additional file 1: Table S1.

The PMTCT service readiness index was then totaled by adding the presence of each indicator, with equal weight given to each of the domains and each of the indicators within the domains. As the target was 100%, each domain accounted for 25% (100%/4) of the index. The percentage for each indicator within the domain was equal to 25% divided by the number of indicators in that domain. The PMTCT service readiness index for each facility was then calculated by summing the percentages. The facility with less or more score in PMTCT readiness index were considered to have lower or higher readiness respectively.

### Controlling (adjusting) factors

These included facilities, provider and client level variables. The facility variables were: facility location categorized as “urban” and “rural;”managing authorityas “public” and “private;” facility type categorized as “hospital,” “health center,” and “dispensary;”quality assurance as “performed” and “not performed;” and routine management meeting as “performed” and “not performed.”The provider variables were sex coded as “male” and “female;” cadre coded as “clinician” and “nurses;” working experience coded as “ ≤ 5 years” and “ > 5 years.” Client variableswere: age coded as “ < 20,” “20–34,” and “ ≥ 35;” and level of formal education coded as “primary,” “secondary,” and “tertiary.”

### Statistical analysis

During descriptive analyses, all categorical variables were summarized using frequencies and percentages and then presented in either tables or graphs. The series of Poisson regression models were fitted to estimate the effect of key independent variable (facility readiness) on the outcome variable (Provision of high-quality of PITC). The Poisson regression models were preferred than the others such as zero inflated and negative binomial models because the response variable did not have excessive number of zeros and did not show over-dispersion respectively. Moreover, the Poisson model uses the log link function that allows all of the predicted values of the outcome variable being non-negative. Initially, unadjusted Poisson regression model (model 1) was fitted to identify controlling (adjusting) variables that would be included for multivariable analysis. All variables with *P* < 0.05 were selected and included in the multiple Poisson regression model (model 2) using a criterion based procedure known as Akaike’s Information Criterion (AIC). Despite AIC use similar approach like backward elimination method, it tends to retain some important variables that needed to be included in the final model. Similar approach was used to perform a sensitivity analysis of each domain of the facility readiness score (staff and guidelines, equipment, diagnostics, and medicine and commodities), to see which domain(s) were most strongly associated with provision of high-quality PITC. A Pearson’s chi-square (χ^2^) test and its corresponding *P* < 0.05 was considered statistically significant. The generalized variance inflation factor (VIF) was performed to test for multicollinearity, which usually should not exceed 5. In this analysis each variable presented with VIF < 2.0, suggesting the absence of multicollinearity in the fitted models. All statistical analyses were performed using STATA 15 (StataCorp, College Station, TX). All estimates were weighted to correct for non- responses and disproportionate sampling. We properly adjusted for clustering observed at provider level by using survey method (“svy” command in STATA) to correct the standard errors for design effect.

### Sensitivity analysis

We disaggregated the key independent variable (facility readiness) into four predetermined domains (staff and guidelines, equipment, diagnostics, and medicine and commodities) and performed the sensitivity analysis to identify which domain had stronger association with provision of high-quality PITC.

## Results

### Baseline characteristics of the observed first-visit ANC consultations

Table 1 presents a summary of the baseline characteristics of the observed first-visit ANC consultations according to the health facilities, health providers, and clients’ characteristics. Out of 1853 included first-visit ANC consultations, about 82% were observed in publicly owned facilities and 68% were observed in lower-level facilities (dispensary or clinics). Less than one-fifth of the observed consultations included clients with secondary or above education level.

**Table 1:** Baseline characteristics of first-visit ANC consultations, Tanzania SPA 2014–2015 (*n* = 1853)

Variable		n(%)
Managing authority		
Public		1519 (81.98)
Private		334 (18.02)
Facility location		
Rural		1383 (74.64)
Urban		470 (25.36)
Facility type		
Clinic and dispensary		1253 (67.62)
Health center		336 (18.13)
Hospital		264 (14.25)
Quality assurance		
Not Performed < 1 year		1375 (74.20)
Performed < 1 year		478 (25.80)
Routine management meeting		404 (62.4)
Not Performed		348 (18.78)
Performed		1505 (81.22)
Provider’s sex (Female)		
Male		309 (16.68)
Female		1544 (88.32)
Cadre		782 (95.1)
Nurses		1744 (94.12)
Clinicians		109 (5.88)
Working experience		392 (47.7)
< 5 years		1057 (57.04)
≥ 5 years		796 (42.96)
Maternal age		
< 20 years		351 (18.94)
20–35 years		1229 (66.34)
> 35 years		273 (14.73)
Maternal education level		
None		430 (23.21)
Primary		1145 (61.79)
Secondary and above		278 (15.00)

### Provision of high-quality of PITC services

**Fig. 2:**
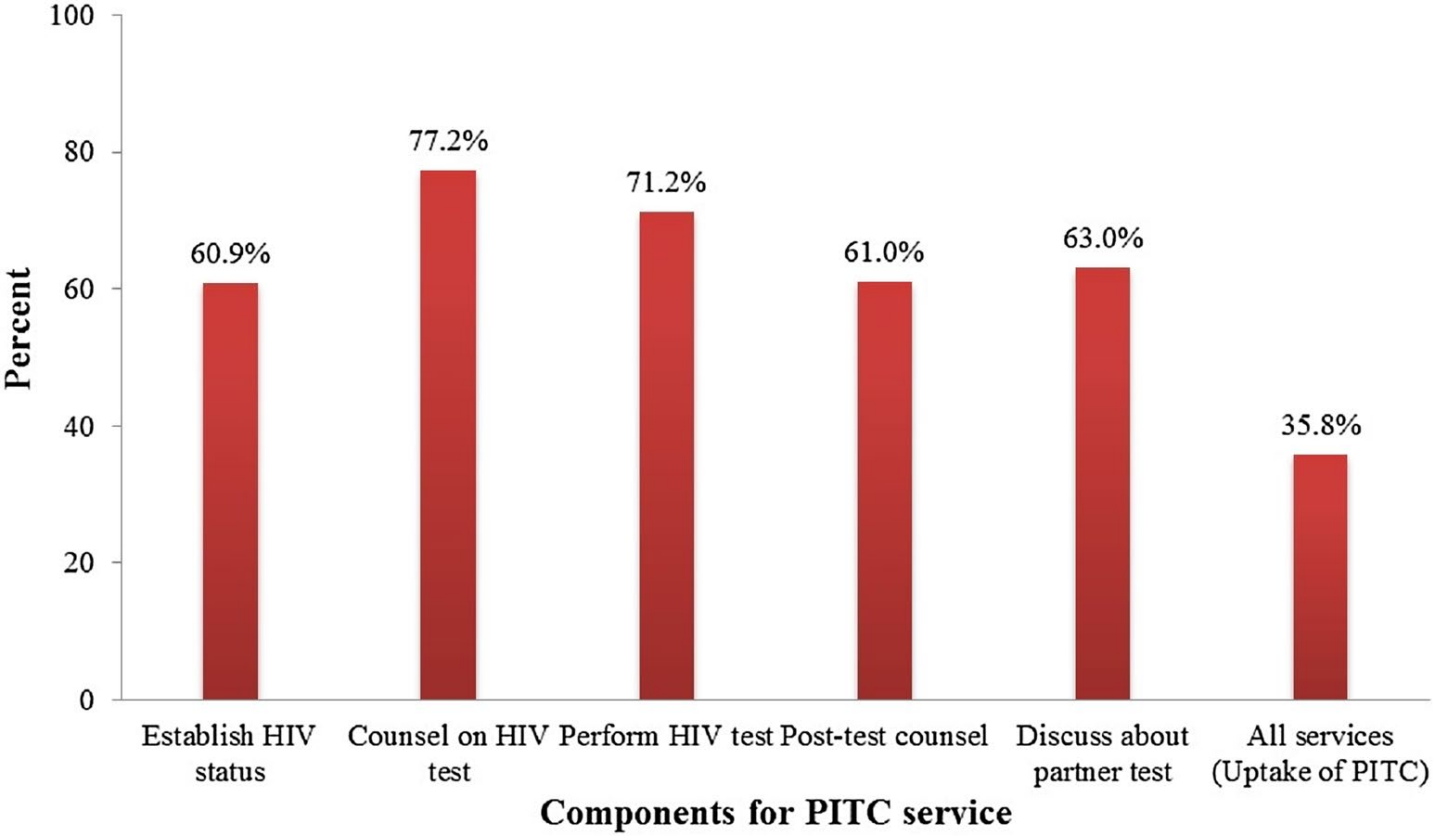
Overall and specific service of PITC during observed first ANC visit, TSPA 2014–2015 (n = 1853)

Figure 2 shows the percentages of the five components used to assess the provision of high-quality of PITC to pregnant women during the first-visit ANC consultations. Although the performance of all five PITC services was relatively low (35.8%), there was a satisfactory proportion of high-quality in some separate individual components such as counseling on HIV tests (77.2%) and performing or referring to HIV tests (71.2%).

### Availability of PMTCT service readiness indicators

Table 2 presents the distribution of PMTCT services readiness indicator. About 80% of the facilities had “PMTCT guidelines” and “visual and auditory privacy” area for PMTCT. More than half of the facilities had observed not having HIV diagnostic capacity for adults. Majority of the facilities had maternal Antiretroviral (76.2%) and Nevirapine syrup (69.1%) while few had Zidovudine syrup (3.7%). The mean percentage of PMTCT readiness score were relatively low 63.96 [61.32–66.59]%.

**Table 2:** Distribution of PMTCT service readiness indicators, TSPA 2014–2015 (n = 648)

Variable	n (%)
Staff and training	
Presence of guidelines for PMTCT	513 (79.17)
Availability of trained staff	385 (59.41)
Equipment	
Presence of visual and auditory privacy	533 (82.25)
Diagnostics	
HIV diagnostic capacity for adults	300 (46.30)
HIV diagnostic capacity for infants	407 (62.81)
Medicines and commodities	
Zidovudine syrup	24 (3.70)
Nevirapine syrup	448 (69.14)
Maternal Antiretroviral	494 (76.23)
The mean PMTCT readiness score	Mean [95% CI]
Facility readiness	63.96 [61.32–66.59]
Total	648

### Association between facility readiness and provision of high-quality of PITC

Table 3 presents the results of unadjusted (model 1) and adjusted (model 2 models that examine the association between facility readiness and provision of high-quality of PITC. The results of the adjusted Poisson model (model 2) shows that, given the other variables are held constant in the model, the provision of high-quality PITC was expected to increase by 7.5% more for each percentage increase of facility readiness score ( β = 0.075, *P* = 0.00).

**Table 3:** Poisson regression analyses for provision of high-quality of PITC and facility readiness unadjusted (Model 1) and adjusted by the selected factors (model 2)

Variable	Model 1	Model 2
	β (Robust SE)	β (Robust SE)
Facility readiness score		
Score	0.069 (0.016)**	0.075 (0.017)**
Managing authority (ref. Public)		
Private	− 0.129 (0.082)*	− 0.123 (0.070)*
Facility location (ref. Rural)		
Urban	− 0.022 (0.060)	
Facility type (ref. Clinic/dispensary)		
Health centre	0.068 (0.054)*	− 0.083 (0.062)*
Hospital	0.053 (0.055)*	− 0.069 (0.073)*
Quality assurance (ref. Not performed < 1 year)		
Performed < 1 year	0.068 (0.055)*	− 0.015 (0.068)
Routine management meeting (ref. Not performed)		
Performed	0.088 (0.099)	
Provider’s sex (ref. Male)		
Female	− 0.034 (0.070)	
Cadre (ref. Nurses)		
Clinicians	0.239 (0.144)*	0.179 (0.114)*
Working experience (ref. < 5 years)		
≥ 5 years	0.026 (0.054)	
Age (ref. < 20 years)		
20–35 years	0.061 (0.052)*	0.059 (0.047)*
> 35 years	0.042 (0.066)*	0.055 (0.062)*
Education level (ref. None)		
Primary	0.005 (0.049)	
Secondary and above	0.047 (0.062)	

### Results of sensitivity analysis

Table 4 presents the summary of sensitivity analysis, which indicates that the provision of high-quality of PITC were expected to increase more for each percentage increase of score in domain of equipment, diagnostics, and medicines and commodities. In addition, in module 5 and 6 the provision of high-quality of PITC tend to decrease by 17.5% in private compared to public facilities.

**Table 4:** Adjusted Poisson regression analyses for provision of high-quality of PITC and domain-specific of facility readiness (Model 3, 4, 5, and 6)

Variable	Model 3	Model 4	Model 5	Model 6
	β (Robust SE)	β (Robust SE)	β (Robust SE)	β (Robust SE)
Facility readiness score				
Score	0.080 (0.041)*	0.165 (0.041)**	0.104 (0.040)**	0.168 (0.048)**
Managing authority (ref. Public)				
Private	− 0.144 (0.081)*	− 0.097 (0.072)*	− 0.175 (0.083)**	− 0.175 (0.084)**
Facility type (ref. Clinic/dispensary)				
Health center	0.020 (0.058)	0.032 (0.054)	− 0.041 (0.063)	0.030 (0.058)
Hospital	0.038 (0.072)	0.068 (0.071)	− 0.035 (0.077)	0.023 (0.075)
Quality assurance (ref. Not performed < 1 year)				
Performed < 1 year	0.033 (0.066)	0.027 (0.064)	0.012 (0.069)	0.027 (0.064)
Cadre (ref. Nurses)				
Clinicians	0.212 (0.131)*	0.200 (0.121)*	0.181 (0.134)*	0.238 (0.128)*
Age (ref. < 20 years)				
20–35 years	0.053 (0.051)*	0.053 (0.050)*	0.064 (0.050)*	0.059 (0.048)*
> 35 years	0.049 (0.063)*	0.055 (0.062)*	0.059 (0.062)*	0.043 (0.062)*

Model 3 for the domain of “Staff and training”

Model 4 for the domain of “Equipment”

Model 6 for the domain of “Diagnostics”

Model 5 for the domain of “Medicines and commodities”

^*^ = p < 0.2, ** = p < 0.05

## Discussion

This study was conducted to examine whether the facility readiness to provide PMTCT services promotes the provision of high-quality PITC to pregnant women during the first-visit ANC. The findings presented in this study indicated moderate average score of facilities readiness to provide PMTCT services and the high-quality of PITC to pregnant women during the first-visit ANC. In addition, the study indicated that the provision of high-quality of PITC to the pregnant women was expected to increase more for each percentage increase of facility readiness score.

The unsatisfactory facility readiness to provide PMTCT services reported in this study indicates the existing challenges to Tanzanian health systems which limit the real-life effectiveness of PMTCT interventions [16], such as shortage of human resources, diagnostic tools, and medicines [26, 27]. This situation hinders the progress towards the EMTCT target across the Global Plan priority countries (22 countries that accounted for 90% of pregnant women living with HIV worldwide) that including Tanzania [28].

Similar to an earlier study conducted in Tanzania [29], more than two-thirds of the observed consultations in this study providers performed HIV tests to the pregnant women. However, the Tanzanian national guideline for PMTCT recommends that every pregnant woman during first-visit ANC should receive all five components of PITC. In this study, only about one-third of pregnant women received all five components required for PITC. This low provision of high-quality of PITC indicates that the majority of pregnant women are not receiving the full package of PITC components. This might compromise the acceptance, adherence, and retention rates for PMTCT interventions because women receive inadequate PITC services during ANC. [30, 31] Previous studies conducted in Tanzania showed that adherence to ART and retention are poorly associated with the readiness indicators listed above which might be expected to work primarily on detection and treatment initiation [32, 33]. Despite the reported shortage of human resources in Tanzanian health systems, the emphases should be made to ANC health providers to consider the comprehensive provision of high-quality of PITC to pregnant women. The provision of inadequate quality of PITC was reported in studies conducted in Ethiopia and Nepal [34, 35].

Provision of PMTCT services can be broadly compromised by health systems factors. Therefore, for the health provider to deliver the high-quality PMTCT services that are accessible, equitable, safe, and responsive to the patients, the high readiness of the facilities is required [36, 37]. This argument is supported by the findings of the current study which indicated a strong association between the facilities with high readiness to provide PMTCT and provision of high-quality of PITC during first-visit ANC in Tanzania. The observed association may be because facility with high readiness is more likely to be committed to providing all PMTTCT services including provision of high-quality PITC services to identify pregnant women with HIV at the early gestational period. Therefore, these facilities tend to have all diagnostic tools, equipment, and medical supplies related to PMTCT and PITC services. In contrast, facilities with low readiness are usually facing inadequate access to important equipment, medical supplies, and trained staff to provide PMTCT and PITC services [27, 38].

The current study had some limitations including the inability to provide a causal connection due to the study design. What has been presented are only associations and should be presented with this word of caution. As the study restricted to the first-visit ANC, the findings may have limited generalizability to all other ANC visits. Although the use of direct observation is regarded as the gold standard, this approach is susceptible to observation biases as well as the Hawthorne effect “the alteration of behaviour by the participants of a study as a result of their awareness of being observed” [39]. Despite the limitations, this is the first study to show the association of PMTCT facility readiness and provision of high-quality of PITC to pregnant women attending the first-visit ANC in Tanzania. Also, the study used a nationally-representative sample with a high response rate and robust sampling procedure, which suggest our findings accurately reflect the current situation about PMTCT and quality of PITC in Tanzanian health systems.

## Conclusion

In summary, even though the majority of pregnant women in Tanzania receives HIV tests during first-visit ANC, only a few of them receives high-quality of PITC services as recommended by WHO. In order to increase high-quality of PITC, efforts should be made to improve the PMTCT facility readiness by increasing availability of trained staffs, diagnostic tools, and ARTs among health facilities in Tanzania.

## Supplementary Information

**Additional file1: Table S1.** Summary of the measurement procedure of key independent variable “Facility readiness.”

## Data Availability

The dataset used for this secondary analysis was generated from the original Tanzanian SPA datasets available in the DHS Program repository: http://dhsprogram.com/data/available-datasets.cfm. The generated dataset is currently stored and accessible by the first author. However, it is available upon request to the first author at the contact address provided in this article.

## Abbreviations

AIDS: Acquired immunodeficiency syndrome
ANC: Antenatal
HIV: Human immunodeficiency virus
MoHCDGEC: Ministry of health, community development, gender, elderly, and children
MTCT: Mother-to-child transmission
PITC: Provider-initiated HIV testing and counseling
PMTCT: Prevention of mother-to-child transmission
SE: Standard error
SPA: Service provision assessment
SSA: Sub-Saharan Africa
WHO: World Health Organization
