# Supplementary material for: Does facility readiness promote high-quality of provider-initiated HIV testing and counseling to pregnant women? A national survey for improving policy of prevention of mother-to-child transmission of HIV in Tanzania
Source: AIDS Res Ther. 2021 Jul 3;18:38. doi: 10.1186/s12981-021-00362-y (PMC8254244; doi:10.1186/s12981-021-00362-y)
Supplement: Supplementary file 1 — Additional file1: Table S1. Summary of the measurement procedure of key independent variable “Facility readiness.” [file 12981_2021_362_MOESM1_ESM.pdf]

S1 Table: Summary of the measurement procedure of key independent variable "Facility readiness"

Domain	Indicators	Measure	Percent score (%)	
			Indicator	Domain
Staff and guidelines	Guidelines for PMTCT	Yes	6.25	25.00
		No	0.00	
	Guidelines for infant and young child feeding counselling	Yes	6.25	
		No	0.00	
	At least one staff trained in PMTCT	Yes	6.25	
		No	0.00	
	At least one staff trained in infant and young child feeding	Yes	6.25	
		No	0.00	
Equipment	Presence of visual and auditory privacy	Yes	25.00	25.00
		No	0.00	
Diagnostics	Presence of RDT or ELISA for HIV testing of adults	Yes	12.50	25.00
		No	0.00	
	Presence of DBS filter paper for diagnosing HIV in newborns	Yes	12.50	
		No	0.00	
Medicine and commodities	Zidovudine syrup	Yes	8.33	25.00
		No	0.00	
	Nevirapine syrup	Yes	8.33	
		No	0.00	
	Maternal ARV prophylaxis	Yes	8.33	
		No	0.00	
Total PMTCT readiness index score				100.00

Note: Presence of maternal ARV were considered based on availability of either Option A: (AZT, NVP, and 3TC) or Option B: (AZT + 3TC + LPV or AZT + 3TC + ABC or AZT + 3TC + EFV or TDF + 3TC (or FTC) + EFV)
